# Pharmacogene Variants Associated with Liver Transplant in a Twelve-Year Clinical Follow-Up

**DOI:** 10.3390/pharmaceutics14020354

**Published:** 2022-02-03

**Authors:** Luis Sendra, Gladys G. Olivera, Rafael López-Andújar, Cristina Serrano, Luis E. Rojas, Eva María Montalvá, María José Herrero, Salvador F. Aliño

**Affiliations:** 1 Pharmacogenetics and Gene Therapy Unit, Instituto de Investigación Sanitaria La Fe, 46026 Valencia, Spain; luis.sendra@uv.es (L.S.); gladyso@alumni.uv.es (G.G.O.); alino@uv.es (S.F.A.); 2 Gene Therapy and Pharmacogenomics Group, Pharmacology Department, Faculty of Medicine, Universitat de València, Av. Blasco Ibáñez 15, Lab. 03, 46010 Valencia, Spain; 3 Unit of Hepatobiliopancreatic Surgery and Transplant, Hospital Universitario y Politécnico La Fe, 46026 Valencia, Spain; rafaellopezandujar@gmail.com (R.L.-A.); montalva.oron@gmail.com (E.M.M.); 4 Spanish Clinical Research Network, SCReN, Instituto de Investigación Sanitaria La Fe, 46026 Valencia, Spain; crisserrano.hrc@gmail.com; 5 Department of Internal Medicine, Faculty of Medicine, Pontificia Universidad Católica de Chile, Santiago 3542000, Chile; lsroja@gmail.com; 6 Program of Pharmacology and Toxicology, Faculty of Medicine, Pontificia Universidad Católica de Chile, Santiago 3543151, Chile; 7 Unit of Clinical Pharmacology, Drugs Area, Hospital Universitario y Politécnico La Fe, 46026 Valencia, Spain

**Keywords:** pharmacogenetics, immunosuppressant, ABC, CYP, SLCO

## Abstract

Some gene polymorphisms have been previously associated individually with tacrolimus efficacy and toxicity, but no long-term study to determine the role of pharmacogene variants in the clinical evolution of liver-transplanted patients has been addressed so far. In the present work, we analyzed the relation between highly-evidenced genetic polymorphisms located in relevant pharmacogenes and the risk of suffering premature death and other comorbidities such as cancer, diabetes mellitus, arterial hypertension, graft rejection, infections and nephrotoxicities in a cohort of 87 patients (8 were excluded due to early loss of follow-up) transplanted at Hospital La Fe in Valencia (Spain) during a 12-year follow-up. Employing a logistic regression model with false discovery rate penalization and Kaplan–Meier analyses, we observed significant association between survival rates and metabolizer genes. In this sense, our results show an association between MTHFR gene variants in donor rs1801133 (HR: 7.90; *p*-value: 0.032) and recipient rs1801131 (HR: 7.34; *p*-value: 0.036) and the group of patients who died during the follow-up period, supporting the interest of confirming these results with larger patient cohorts. In addition, donor polymorphisms in *UGT1A9* metabolizer gene rs6714486 (OR: 0.13; *p*-value: 0.032) were associated with a lower risk of suffering from de novo cancer. Genetic variants in *CYP2B6* metabolizer gene rs2279343 demonstrated an association with a risk of infection. Other variants in different locations of *SLCO1A2*, *ABCC2* and *ABCB1* transporter genes were associated with a lower risk of suffering from type 2 *diabetes mellitus*, chronic and acute nephrotoxicities and arterial hypertension. Results suggest that pharmacogenetics-derived information may be an important support for personalized drug prescription, clinical follow-up and the evolution of liver-transplanted patients.

## 1. Introduction

In 2019, over 39,000 liver transplantations were performed worldwide [1]. Post-transplantation survival rate has continuously increased during the past decade to achieve 83% at 1 year, 71% at 5 years, 61% at 10 years, 51% at 15 years and 41% at 20 years [2] thanks to improved therapeutic follow-up of patients. However, transplanted patients have a 30% higher overall risk of exitus than healthy patients do. Furthermore, these patients frequently present comorbidities [3], such as tumors, arterial hypertension, diabetes mellitus, organ rejection, infections and acute and chronic nephrotoxicities, which worsen their prognosis and welfare and generate a huge increase in healthcare spending. These clinical variables widely differ among patients, and whereas some of them have high survival rates over 10 years without clinical problems, others present several comorbidities, with frequent hospitalizations and reduced survival probability. The reasons underlying the different clinical evolution after liver transplantation are not completely elucidated, although it is thought that the distinct patients response to the transport and metabolism of the immunosuppressant drugs employed plays an important role [4]. Many different proteins are involved in drug processing, and these are encoded by pharmacogenes [5,6,7,8], which, as other genes, present natural variability among the population. Some of these genetic variants affect the normal function of some drugs and can result in deleterious effects on patients, and this is especially relevant in the liver transplant setting due to its pivotal role in drug metabolism and detoxification [9]. To date, only variants of *CYP3A5* gene have achieved the top level of scientific evidence quality (1A) regarding tacrolimus [10,11,12], the most widely employed immunosuppressant, whereas genetic variants of *CYP3A4* gene reached high [13,14], not top, quality (level 1B). In this regard, FDA includes *CYP3A* pharmacogenetics information in tacrolimus drug label. To date, most previous studies have focused on these genes and short follow-up periods. However, patients receive multiple drugs during their entire life, and some information, such as interactions or metabolism pathway saturation, could be overlooked. In the present work, we have developed a highly evidenced [15] SNPs panel that includes variants affecting transport, metabolism and signaling pathway genes involved in immunosuppressants and other prescribed drugs. Liver transplantation is a special setting given its pivotal role in drug metabolism and detoxification, as well as the fact that two genotypes (from the recipient and the donor) are participating [16]. Some drug transporters are encoded by the recipient genome, but key proteins in liver metabolism are encoded by the donor genotype, and these two DNA sequences could be different. For these reasons, genotyping both the donor and the recipient is required.

**Figure 1:**
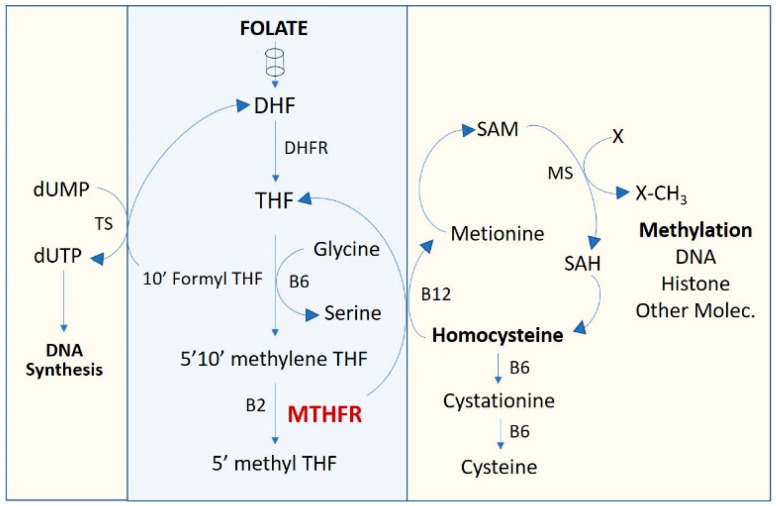
One-carbon metabolism pathway. DHF, dihydrofolate; DHFR, dihydrofolate reductase; dTMP, deoxythymidine monophosphate; dUMP, deoxyuridine monophosphate; MTHFR, methylenetetrahydrofolate reductase; SAM, S-Adenosyl methionine; THF, tetrahydrofolate.

In the present work, DNA samples from donors and recipients of 87 liver transplantations (8 were excluded later due to early, less than 1 year, loss of follow-up) performed at Hospital La Fe (Valencia, Spain) during 2008 and 2009 were genotyped for the selected SNPs. The association of gene variants with clinical results during the 12-year clinical follow-up was evaluated. The goal of the present work was to evaluate the association between genetic variants of pharmacogenes and clinical long-term evolution of liver-transplanted patients. We studied both complex multi-factorial clinical events with diverse etiology, such as cancer and survival, and other events that could be more directly associated with a cause. Accordingly, the data analysis was addressed in two ways: (a) an analysis based on the hypothesis of a possible association of a subgroup of genes with a higher risk of cancer or lower survival. It has been reported that *MTHFR* variants with reduced gene function (rs1801131 and rs1801133) affect the folate cycle (Figure 1), which has a pivotal role in cell metabolism, and provoke important oncologic [17] and non-oncologic [18] clinical manifestations; (b) in the absence of hypothesis, the association of specific clinical parameters with all the variants of the pharmacogenes used in our panel was evaluated.

## 2. Methods

### 2.1. Patients

According to the Declaration of Helsinki, the study was approved by the Institutional Ethics Board of the Hospital Universitari i Politècnic La Fe, Valencia, Spain. A written informed consent was obtained from all the subjects included in this study. All liver-transplanted adult patients (>18 years) operated on in 2008 and 2009 and treated with tacrolimus were assessed for potential study inclusion. The demographic, clinical and laboratory data were obtained from electronic health medical records (Table 1) under the approval of the Hospital Ethics Committee (references: 2021-370-1).

### 2.2. Immunosuppressive Therapy

Tacrolimus (Prograf^®^, Astellas Pharma Inc., Valencia, Spain) was first administered orally within 24 h after surgery, unless there were any clinical complications, at an initial dose of 0.1 mg/kg/day, twice daily. The dosage was adjusted to achieve the target trough level, set between 10 and 15 ng/mL during the first 3 months, 5 and 15 ng/mL until 12 months and 5 and 10 ng/mL thereafter. Methylprednisolone (10 mg/kg) was intravenously administered during surgery. Then, the dosage was gradually reduced and was changed to orally administered prednisolone 1 week after surgery. The prednisolone dosage was gradually diminished and discontinued when clinically possible after the transplant.

Mycophenolate mofetil (MMF) was also administered to diabetic patients who received low doses of steroids, patients with hepatitis C virus infection (HCV) and those with early kidney failure during calcineurin inhibitor therapy. MMF was administered orally at a fixed dose of 1–1.5 g/day. Azathioprine (1.2 mg/kg/day) was used in patients with autoimmune hepatitis or primary biliary cirrhosis. Recipients with acute kidney failure received two 20 mg doses of IV basiliximab (Novartis, East Hanover, NJ, USA) within 6 h post transplantation and on day 4 after transplantation.

### 2.3. Clinical Outcomes

For tumor incidence, only those patients without hepatocellular carcinoma at surgery who had a de novo tumor during the follow-up period were considered. Acute rejection was determined by liver biopsy, and the histologic diagnosis was performed according to Banff criteria. *Diabetes mellitus* and arterial hypertension were diagnosed as usually done in clinical setting. As infections, we considered de novo infections suffered by a transplanted patient. Acute nephrotoxicity was defined as increased serum creatinine level greater than 0.5 mg/dl or 20–25% above the pre-transplant baseline coinciding with high tacrolimus blood levels that decrease with tacrolimus reduction, and excluding other conditions that cause renal impairment such as fever, infection, graft swelling or tenderness, oliguria, bleeding, dehydration, increased resistive index on Doppler ultrasonography or ureteral obstruction. Chronic nephrotoxicity was diagnosed based on clinical criteria: creatinine clearance <60 mL/min for at least 3 months, not coincidental with other above-mentioned clinical parameters causing renal impairment or use of other nephrotoxic drugs. Creatinine clearance was calculated using the Modification of Diet in Renal Disease Study Equation (4) [19].

### 2.4. Single Nucleotide Polymorphism Identification

The genotyping of the SNPs was performed using the MassARRAY platform by SEQUENOM (currently Agena Bioscience, San Diego, CA, USA) at the SCSIE (Central Service of Experimental Research Support) at the Faculty of Medicine, Universitat de Valencia, Spain. In brief, genomic DNA was extracted from whole blood of transplanted recipients and donors, collected in EDTA tubes. The DNA was purified from 200 μL of blood using a commercially available kit (Ultra Clean Blood Spin DNA Isolation Kit; MoBio Laboratories Inc., Carlsbad, CA, USA) following the manufacturer’s instructions. After quantification using a spectrophotometer (NanoDrop Technologies Inc., Wilmington, DE, USA) to determine the concentration and purity, DNA was preserved at −20 °C until further use. All the samples were then genotyped in triplicates to assess quality of the technique. Genotype and allele frequencies, MAF and chi-square for Hardy–Weinberg equilibrium determination of all gene variants evaluated are described at Table S1 of Supplementary Materials.

### 2.5. SNPs Panel

SNPs evaluated (Table 2) were selected based on previous studies associating genes with clinical outcomes in liver-transplanted patients, with the addition of other polymorphisms located in genes encoding drug transporters and other signaling pathways with potential interest.

### 2.6. Statistical Analyses

Statistical analyses were performed using SPSS (version 26; IBM; New York, NY, USA) and RStudio (version 1.4.1106; Boston, MA, USA) software. Multivariate logistic regression was employed to evaluate the association of genetic variants with the risk of exitus and tumor incidence during the follow-up period. All gene variants were included as variates; age, gender, hepatitis, hepatocellular carcinoma and cirrhosis were included as co-variates. We calculated hazard ratio (HR) and 95% confidence interval (CI) for clinical outcomes in subjects with all variants to evaluate associations. Survival and tumor logistic regression was performed in a step-wise fashion with SPSS software with false discovery rate penalty, employing those *p*-values obtained for gene variants associated with logistic and Cox regression tests obtained in both exitus and tumor. A *p*-value < 0.05 was considered statistically significant. The time evolution of exitus and cancer prevalence during the follow-up with the significant gene variants selected by logistic regression and FDR was represented by Kaplan–Meier curves, designed by ggplot package in RStudio software. Significance was determined by log-rank analysis. The association of pharmacogene variants with the other clinical variables was evaluated with RStudio software by logistic regression and elastic net fitting (alpha: 0.5; lambda: min; 500 iterations). Elastic net in the fitting of linear or logistic regression models is a regularized regression method that linearly combines the L1 and L2 penalties of the lasso and ridge methods. It is a variable selection method that builds predictive models including only variables with statistical predictive power. It is especially suited for sets of data with many more variables than observations, and it does not produce *p*-values. Variables selected in the model are statistically relevant. Those variables that are not selected are excluded. Elastic net does not calculate either confidence intervals or *p*-values; it only generates predictive models, not inferential ones [20,21]. For those pharmacogenes variants associated with clinical events, Fisher and chi-square contingency analyses were performed depending on the presence of two or three possible genotypes, respectively. This permitted reconfirming the strongest associations.

## 3. Results

### 3.1. Patients’ Genotypes

The genotypic and allelic frequencies of all SNPs evaluated from donors and recipients are shown in Table S1 (Supplementary Materials). MAF and chi-square to determine their Hardy–Weinberg equilibrium were also included.

### 3.2. Survival

The relation between pharmacogene variants and survival was evaluated. We determined the role of genetics in survival at the end of the 12-year study by multivariate logistic regression (Table 3). All SNP variants were included as variables, and age, gender, hepatitis and hepatocarcinoma were used as co-variables. In this study, false discovery rate was applied considering all *p*-values of the variants selected in order to penalize *p*-values and determine the association. Logistic regression with FDR penalty showed that only the CC variant in *MTHFR* rs1801131 for the recipient and the TT variant in *MTHFR* rs1801133 for the donor were significantly (*p* = 0.036 and *p* = 0.032, respectively) related to higher mortality, with 7.34 and 7.90 odds ratios, respectively.

**Figure 2:**
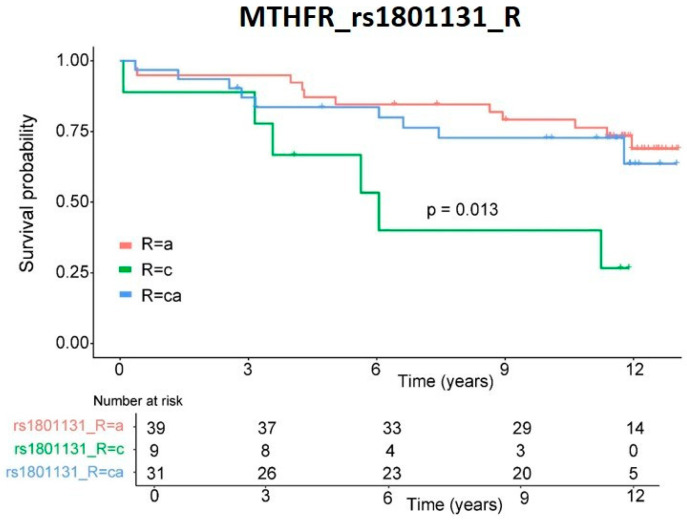
Survival rate. Kaplan–Meier representation of patients’ survival rates depending on genetic variants in recipient *MTHFR* rs1801133 SNP. Significance was obtained by log-rank analysis.

The individual role of these variants on patients’ survival was graphed as a Kaplan–Meier curve (Figure 2), and significance was measured by log-rank. Only rs1801131 SNP demonstrated significant (*p* = 0.013) differences in survival rate.

### 3.3. Tumor

The relation between genotype and post-transplantation tumor incidence risk was also evaluated. As with survival, we studied this relation at the follow-up endpoint by multivariate logistic regression. Statistical analysis included all SNP variants from the donor and recipient as variables and gender, age, hepatitis and hepatocarcinoma as co-variables. After FDR *p*-value correction employing *p*-values of the different gene variants selected, only the TA variant in *UGT1A9* rs6714486 of the recipient (Table 4) was significantly (*p* = 0.032) related to lower tumor probability (HR: 0.13).

**Figure 3:**
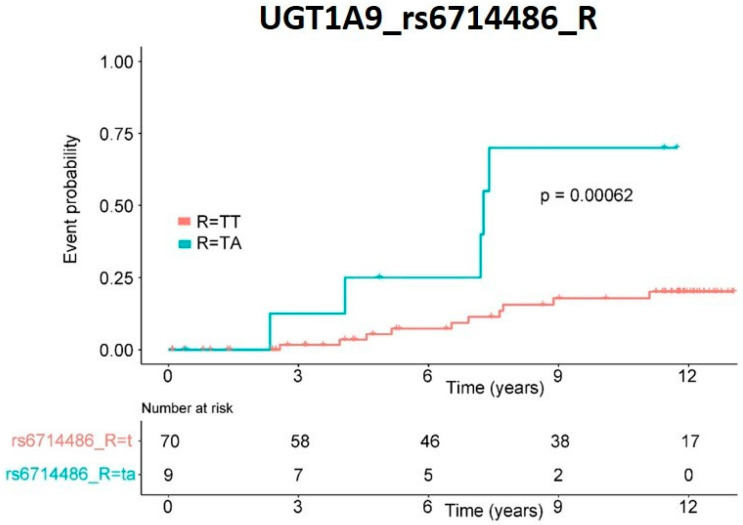
De novo cancer risk. Kaplan–Meier representation of patients’ risk of cancer de novo appearance depending on genetic variants in recipient *UGT1A9* rs6714486 SNP. Significance was obtained by log-rank analysis.

The incidence of tumors over time was represented as a Kaplan–Meier curve (Figure 3). This variant also proved statistically significant (*p*-value = 0.00062) when analyzed individually by log-rank.

### 3.4. Other Clinical Variables

Other clinical variables commonly associated with liver-transplanted patients and their pharmacological treatment, including arterial hypertension, *diabetes mellitus*, organ rejection, infections and acute and chronic nephrotoxicity, were also evaluated by logistic regression. Since these pathologies are also present in the general population of similar age, we employed a more restrictive statistical analysis, logistic regression with elastic net penalty fitting, to exclude potential bias and ensure the assessment of pharmacogene variant association. All SNP genotypes were included as variates, and gender and age as co-variates. For selected variables, contingency tests (Fisher or chi-square) were performed to calculate their *p*-values and determine the differences between groups. When including all SNPs as variables, we did not find significant association of pharmacogene variants with organ rejection, arterial hypertension or acute nephrotoxicity. However, the results (Table 5) reported that the CA variant in gene *SLCO1A2* rs11568563 of the recipient was statistically associated with a lower risk (OR: 0.705; *p*-value = 0.002) of suffering from *diabetes mellitus*. Regarding chronic nephrotoxicity, which affected 30% of our cohort, statistical analysis determined that two variants in *ABCC2* were significantly associated with a reduced risk of suffering from this pathology. These variants were CT in rs374066 and TC in rs717620, both in the recipient, which presented ORs of 0.920 (*p*-value = 0.017) and 0.878 (*p*-value = 0.012), respectively. On the other hand, the GA genotype in rs2279343 of *CYP2B6* gene in the recipient was selected as a predictive variable by elastic net and associated with a higher risk of infection (OR: 1.116; *p*-value = 0.086).

In order to determine the role of the gene variants depending on their function, the SNPs located in genes encoding transporter proteins (ABC and SLCO) and in metabolizer and signaling pathway proteins (CYP, UGT, TPMT, MTHFR, NOD2) were separately re-evaluated. With this analysis, fewer variates were included, and thus the penalty applied was lower. These results permitted observing more associations.

### 3.5. Other Clinical Variables: Transporter Genes

In *diabetes mellitus* (Table 6), the association of CA variant in rs11568563 (*SLCO1A2*) of the recipient was confirmed, and OR was even lower (0.550; *p*-value = 0.002). In this sense, whereas patients with the AA variant (n: 64) in this SNP were nearly distributed normally in the presence (n: 27) or absence (n: 37) of disease, none of the patients with the CA variant (n: 15) presented *diabetes mellitus* during the 12-year post-transplantation period. Besides this SNP, the CA variant in rs2231142 of gene *ABCG2* of the recipient was associated with a slightly higher (OR: 1.008; *p*-value = 0.082) risk of DM2. Two variants in two SNPs of *ABCB1* gene in the donor were related with DM2; the CT variant in rs1128503 had a lower risk (OR: 0.922; *p*-value = 0.122), and the TT variant in rs2032582 presented a slightly higher risk (OR: 1.062; *p*-value = 0.133), none of them being significant after contingency analysis.

In arterial hypertension, we found associations of variants in *ABCB1* and *ABCC2* genes of the recipient with lower risk. In *ABCB1* gene, the TT variant in rs1045642 (*p*-value = 0.017) and rs1128503 (*p*-value = 0.054) and the GG variant in rs229109 (*p*-value = 0.053) reported odds ratios of 0.976, 0.859 and 0.857, respectively. In *ABCC2*, the GA variant in rs2273697 (*p*-value = 0.103) showed an OR of 0.942. Only the TT variant in *ABCB1* rs1045642 of the donor presented association (*p*-value = 0.040) with a decreased risk of suffering from acute nephrotoxicity, with an odds ratio of 0.916. In chronic nephrotoxicity, the same *ABCC2* gene variants observed with the whole panel proved significant but with even lower risk (OR 0.831; *p*-value = 0.017) for rs374066 in the recipient, as well as 0.784 OR (*p*-value = 0.012) in the TC variant in rs717620 of the recipient.

### 3.6. Other Clinical Variables: Metabolizer and Signaling Pathway Genes

Only the GA variant in rs2279343 of recipient *CYP2B6* gene demonstrated an association with higher risk of infections (Table 7), as observed in the whole panel study, though the OR was 1.240. When a contingency test was performed, the *p*-value did not prove significant (*p*-value = 0.086). No other variant had an association with any clinical variable studied.

## 4. Discussion

We have evaluated possible associations between genetic variants on a panel designed to include the SNPs with significant scientific evidence and liver-transplanted patients’ clinical evolution during follow-up 12 years following the intervention. The panel included 37 SNPs from 14 genes: five transporters (18 SNPs), seven metabolizers (14 SNPs) and two signaling-pathway-related (5 SNPs). Differing from other studies, the aim of this work was not to determine direct drug–SNP associations but to analyze the role of pharmacogene variants in patients’ clinical evolution, considering that, besides the immunosuppressant treatment, patients are submitted to other medicines and environmental factors, such as xenobiotics. In our cohort, we found that survival rate was associated with gene variants in *MTHFR*, and cancer incidence risk was associated with genotype in *UGT1A9* metabolizer gene. Other clinical parameters, except for infections, were associated mainly with transporter genes. The associations appeared both in pharmacogenes previously associated with drugs prescribed in transplantation settings and in other locations not previously reported. No variant in *CYP3A4* and *CYP3A5* genes (associated by other authors with tacrolimus) demonstrated significant association with clinical events in our study. In our results, genetic variants affecting transporter genes showed more significant associations with the clinical events studied.

Associations between survival outcome and genotype in pharmacogenes were found. Variants in *MTHFR* (methylenetetrahydrofolate reductase) metabolizer gene, rs1801131 and rs1801133, demonstrated association with a significantly lower survival rate. Although both *MTHFR* rs1801131 and rs1801133 variants showed significant association with exitus altogether when analyzed by multivariate logistic regression with the other genetic variants, rs1801133 did not show significant association with survival rate by the Kaplan–Meier method since this test analyzes the individual role of a variant with the clinical event probability over time. *MTHFR* plays a key role in folate metabolism and DNA synthesis and participates in the pharmacodynamics of several drugs. The one-carbon metabolic pathway (Figure 1) is mainly involved in supplying the metabolites required for DNA synthesis (folate cycle), as well as cell methylation (cysteine cycle) required for the epigenetic regulation of DNA and histones, homocysteine metabolism and serine catabolism, which contribute to the re-methylation of homocysteine to methionine. The activity of MTHFR enzyme must determine the magnitude of folate product supply to the different cycles of the one-carbon metabolic pathway. Thus, the increase of blood homocysteine associated with functional deficiency of MTHFR affects the joints and cognitive capacity and increases the risk of heart disease [22], stroke, hypertension, embolism and other neurological signs. On the other hand, the deficient activity of MTHFR reduces the amount of required precursors for DNA synthesis, exerting a synergistic effect with methotrexate in rheumatic diseases [23]. This has been associated with unfavorable evolution in patients with neuroblastoma [24]. It has been suggested that a dietary supply of the precursors of these cycles could be beneficial in patients with deficient MTHFR [18,25], but this remains to be clarified.

In our study, the TA variant in *UGT1A9* was associated with a lower incidence (HR: 0.13) of de novo cancer. When the time of tumors’ appearance was analyzed individually by Kaplan–Meier for this SNP, it also proved significant. The association of *UGT1A9* rs6714486 with lower cancer incidence in our study coincides with the data of other authors that recently correlated this variant with mycophenolate mofetil efficacy [26].

Regarding our results in diabetes mellitus, hypertension, nephrotoxicities and infections, large limitations were found with regard to proposing a biological mechanism that explains the associations. For this reason, we first relate the associations found with those previously described by other authors. In this sense, variants in up to four different SNPs in *ABCB1* gene have been associated with *diabetes mellitus*, arterial hypertension and acute nephrotoxicity. The same SNPs observed in this study have been associated by other authors [27,28] with different responses to a wide range of medicines, among which cyclosporine, tacrolimus, everolimus, glucocorticoids, sirolimus and many other habitual drugs used in organ transplantation settings are included. We also found significant associations between variants present in *ABCC2* gene (rs2273697 GA, rs717610 CT and rs3740066 TT) and clinical events, but protective ones in this case, such as a reduced risk of arterial hypertension and a significantly lower incidence of chronic nephrotoxicity. Variants in this gene have already been related by other authors to metabolism and pharmacokinetic issues of immunosuppressant drugs such as mycophenolic acid and tacrolimus [29,30,31]. All the SNPs in *ABC* genes described in the present work had already been associated with tacrolimus toxicity and efficacy issues in organ transplantation settings except for *ABCG2* gene and rs717620 of *ABCC2* gene. Variants in *ABCB1* and *ABCC2* genes, encoding transporters located in the apical side of tubular kidney cells, have been associated with nephrotoxicity caused by intracellular drug accumulation [32] due to an imbalanced efflux. Despite all of them presenting low levels of evidence, the data reported by other authors and the present work shed light on the importance of genetic variability in *ABC* superfamily genes. The importance of transporter genes and their variants in drug management and patients’ health becomes manifest, as other genetic variants present in *SLCO* superfamily genes are associated with DM2 incidence (*SLCO1A2*). In our cohort, the presence of CA variant in *SLCO1A2* rs11568563 has been strongly associated with a lower risk of DM2 incidence. These results reinforce the observations supporting the idea that gene variants in *SLCO1A2* could be involved in the pathogenesis of metabolic syndrome, characterized by an increased incidence of DM2 [33].

Regarding *CYP2B6*, a higher risk of infection was observed in patients with a *CYP2B6* metabolizer gene variant (rs2279343 GA). Although the G variant in rs2279343 has been previously correlated with efavirenz [34], methadone, bupropion, nicotine and others [35], no previous studies reported an association with immunosuppressant drugs. We do not know the reasons that could justify its association with infections. However, some publications relate variants in this gene to the activity of the immune system, albeit with little evidence at this moment [36].

## 5. Conclusions

A panel of pharmacogene variants, including high-evidenced SNPs, permitted us to observe their associations with clinical events in liver-transplanted patients during 12-year follow-up. We observed associations between exitus and genetic variants in *MTHFR* metabolizer gene, as well as between a decreased risk of de novo cancer and variants in *UGT1A9* metabolizer gene. In addition, genetic variants in *CYP2B6* metabolizer gene proved association with higher risk of infections. On the contrary, gene variants in *SLCO1A2*, *ABCB1* and *ABCC2* transporter genes were associated with a lower risk of suffering from type 2 *diabetes mellitus*, arterial hypertension and acute and chronic nephrotoxicities. Our results suggest that early pharmacogenetics analyses could help clinicians to personalize their pharmacological strategies during patient follow-ups, aiming to improve patients’ survival and quality of life. Further studies will be required to confirm the interest in deepening the exploration of these variants to achieve more robust evidence.

## Figures and Tables

**Table 1:** Patients’ demographics.

Gender (*n*)	Average ± SD	%
Male (m)	55	69.62
Female (f)	24	30.38
Weight (kg)	74.90 ± 13.10	
Age at Tx (years)	54.65 ± 10.24	
Diagnosis at Tx (*n*)		
Cirrhosis	70	88.61
Hepatitis C virus (HCV)	37	46.84
Hepatocellular carcinoma (HCC)	31	39.24
Tacrolimus dose (mg/kg/day)	0.09 ± 0.02	
Hospital stay (days)	24.14 ± 43.07	
Retransplantation required (*n*)	5	6.33
Exitus during follow-up		
*n*	26	32.91
Time (years)	9.22 ± 3.97	
De novo cancer during follow-up		
*n*	15	18.99
Time (years)	6.21 ± 2.40	
Clinical events during follow-up (*n*)		
De novo DM2	27	34.18
De novo arterial hypertension	29	36.71
Graft rejection	36	45.57
Infections	46	58.23
Acute nephrotoxicity	28	35.44
Chronic nephrotoxicity	24	30.38
Patients with emergencies	58	73.42
Average emergencies	7.17 ± 9.49	
Patients requiring hospitalizations	68	86.08
Average hospitalizations	4.81 ± 4.15	
Pharmacological treatment		
Tacrolimus	79	100.00
Micophenolic acid	36	45.57
Corticosteroids	75	94.94
Time (months)	11.00 ± 9.30	
Azatioprin	12	15.19
Induction therapy	4	5.06
Nephrotoxic drugs	11	13.92
CYP3A5 modifier drugs	5	6.33

**Table 2:** SNPs included within the pharmacogenetics panel.

Gene	Function	SNP	
*ABCB1*	Transporter	rs1045642	rs2235013
		rs1128503	rs2235033
		rs2032582	rs3213619
		rs229109	rs9282564
*ABCC2*	Transporter	rs3740066	rs717620
		rs2273697	
*ABCG2*	Transporter	rs2231137	rs2231142
*CYP2B6*	Metabolizer	rs2279343	rs3745274
*CYP2C19*	Metabolizer	rs4244285	
*CYP2C9*	Metabolizer	rs1799853	rs1057910
*CYP3A4*	Metabolizer	rs2740574	
*CYP3A5*	Metabolizer	rs41303343	rs776746
		rs10264272	
*MTHFR*	Metabolizer	rs1801131	rs1801133
*NOD2*	Signaling pathway	rs2066844	rs2066845
*SLCO1A2*	Transporter	rs11568564	rs72559749
		rs11568563	
*SLCO1B1*	Transporter	rs2306283	rs4149056
*TPMT*	Signaling pathway	rs1142345	rs1800462
		rs1800460	
*UGT1A9*	Metabolizer	rs17868320	rs72551330
		rs6714486	

**Table 3:** Pharmacogene variants significantly associated with survival rates after FDR calculation employing the *p*-values of selected variables in exitus and cancer risk studies by logistic and Cox regression.

				Model Data					
				Logistic Regression				CI (95%)	
Gene	SNP	D/R	Genotype	R^2^ Cox Snell	R^2^ Nagelkerke	*p*-Value	OR	Lower	Upper
*MTHFR*	rs1801131	R	CC	0.294	0.409	0.036	7.34	1.39	38.70
*MTHFR*	rs1801133	D	TT			0.032	7.90	1.67	37.43

SNP: single nucleotide polymorphism; D: donor; R: recipient; OR: odds ratio; CI: confidence interval.

**Table 4:** Pharmacogene variants significantly associated with tumor incidence after FDR calculation employing the *p*-values of selected variables in cancer risk and exitus studies by logistic and Cox regression.

				Model Data					
				Logistic Regression				CI (95%)	
Gene	SNP	D/R	Genotype	R^2^ Cox Snell	R^2^ Nagelkerke	*p*-Value	OR	Lower	Upper
*UGT1A9*	rs6714486	R	TA	0.248	0.399	0.032	0.13	0.030	0.583

SNP: single nucleotide polymorphism; D: donor; R: recipient; OR: odds ratio; CI: confidence interval.

**Table 5:** Pharmacogene variants associated with other clinical variables selected by multivariate logistic regression and regularized by elastic net statistical analysis. Full panel with all SNPs.

All SNPs							
Clinical Variables	Gene	SNP	D/R	Genotype	*n*	%	OR
*Diabetes mellitus*	*SLCO1A2*	rs11568563	R	CA ^†^	15	18.99	0.705
Infections	*CYP2B6*	rs2279343	R	GA	35	44.30	1.116
Chronic nephrotoxicity	*ABCC2*	rs3740066	R	CT ^†^	39	49.37	0.920
		rs717620	R	TC ^†^	29	36.71	0.878

SNP: single nucleotide polymorphism; D: donor; R: recipient; OR: odds ratio; *n*: number of patients with the indicated genotype; % proportion of patients with the indicated genotype within the population. † significant *p*-value (<0.05) after contingency test analysis.

**Table 6:** Transporter pharmacogene variants associated with clinical variables selected by multivariate logistic regression and regularized by elastic net statistical analysis.

Transporter Genes SNPs					De Novo Disease (*n*)		
Clinical Variables	Gene	SNP	D/R	Genotype	Absence	Presence	OR
*Diabetes mellitus*	*SLCO1A2*	rs11568563	R	A	37	27	-
				CA ^†^	15	0	0.550
	*ABCG2*	rs2231142	R	C	48	21	-
				CA	4	6	1.008
	*ABCB1*	rs1128503	D	C	15	12	-
				CT	28	8	0.922
				TT	9	7	-
		rs2032582	D	GT	28	10	-
				G	20	11	-
				TT	4	6	1.063
Arterial hypertension	*ABCB1*	rs1045642	R	TC	27	15	-
				CC	7	11	-
				TT ^†^	16	3	0.976
		rs1128503	R	CT	29	20	-
				CC	9	8	-
				TT ^†^	12	1	0.859
		rs229109	R	GA	1	4	-
				AA	2	3	-
				GG ^†^	47	22	0.857
	*ABCC2*	rs2273697	R	GG	29	23	-
				AA	3	2	-
				GA	18	4	0.942
Acute nephrotoxicity	*ABCB1*	rs1045642	D	CC	11	10	-
				TC	27	17	-
				TT ^†^	13	1	0.916
Chronic nephrotoxicity	*ABCC2*	rs3740066	R	CC	15	12	-
				CT ^†^	33	6	0.831
				TT	7	6	-
		rs717620	R	CC	27	20	-
				TC ^†^	26	3	0.784
				TT	2	1	-

SNP: single nucleotide polymorphism; D: donor; R: recipient; OR: odds ratio; † significant *p*-value (<0.05) after contingency test analysis.

**Table 7:** Pharmacogene variants associated with other clinical variables selected by multivariate logistic regression and regularized by elastic net statistical analysis. Metabolizer and target pharmacogenes.

Metabolizer and Target Genes SNPs					De Novo Disease (*n*)		
Clinical Variables	Gene	SNP	D/R	Genotype	Absence	Presence	OR
Infections	*CYP2B6*	rs2279343	R	AA	18	18	-
				GA	8	27	1.240
				GG	6	2	-

SNP: single nucleotide polymorphism; D: donor; R: recipient; OR: odds ratio.

## Data Availability

The data presented in this study are available on request from the corresponding author. The data are not publicly available due to privacy and ethical reasons.

## Supplementary Materials

The following are available online at https://www.mdpi.com/article/10.3390/pharmaceutics14020354/s1, Table S1: Patients’ genotype and allele frequencies. Chi-square calculation for Hardy-Weinberg equilibrium comparing the observed vs. the expected genotype frequencies was also calculated.

## Abbreviations

A	Adenine
ABC	ATP-binding cassette
C	Cytosine
CI	Confidence Interval
CYP	Cytochrome P450
DHF	Dihydrofolate
DHFR	Dihydrofolate reductase
DM	Diabetes mellitus
DNA	Deoxyribonucleic acid
dTMP	Deoxythymidine monophosphate
dUMP	Deoxyuridine monophosphate
EDTA	Ethylenediaminetetraacetic acid
FDA	Food and Drug Administration
FDR	False Discovery Rate
G	Guanine
HCV	Hepatitis C virus
MAF	Minor Allele Frequency
MTHFR	Methylenetetrahydrofolate reductase
NOD	Nucleotide-binding oligomerization domain
OR	Odds Ratio
SAM	S-Adenosyl methionine
SLCO	Solute Carrier
SNP	Single nucleotide polymorphism
T	Thymine
THF	Tetrahydrofolate
TPMT	Thiopurine methyltransferase
UGT	Uridine diphosphate glucuronyltransferase
