# Supplementary material for: Pharmacogene Variants Associated with Liver Transplant in a Twelve-Year Clinical Follow-Up
Source: Pharmaceutics. 2022 Feb 3;14(2):354. doi: 10.3390/pharmaceutics14020354 (PMC8878556; doi:10.3390/pharmaceutics14020354)
Supplement: Supplementary file 1 [file pharmaceutics-14-00354-s001.zip › pharmaceutics-1458149-SI.pdf]

pharmaceutics

MDPI

Supplementary Materials: Pharmacogene Variants Associated with Liver Transplant in a Twelve-Year Clinical Follow-Up

Luis Sendra, Gladys G. Olivera, Rafael López-Andújar, Cristina Serrano, Luis E. Rojas, Eva María Montalvá, María José Herrero and Salvador F. Aliño

Table S1. Patients' genotype and allele frequencies. Chi-square calculation for Hardy-Weinberg equilibrium comparing the observed vs the expected genotype frequencies was also calculated.

Gene	SNPs	Genotype	Frequency in Recipients (%)	Allele frequencies	χ^2	Frequency in Donors (%)	Allele frequencies	χ^2
ABCB1	rs1045642	CC	22.78	(C) 49.37†	0.40	26.58	(C) 54.43	1.51
		TC	53.16	(T) 50.63		55.70	(T) 45.57†	
		TT	24.05			17.72		
ABCB1	rs1128503	CC	21.52	(C) 52.53	5.94	34.18	(C) 56.96	0.50
		TC	62.03	(T) 47.47†		45.57	(T) 43.04†	
		TT	16.46			20.25		
ABCB1	rs2032582	GG	29.11	(G) 56.96	1.85	39.24	(G) 63.29	0.12
		GT	55.70	(T) 43.04†		48.10	(T) 36.71†	
		TT	15.19			12.66		
ABCB1	rs229109	AA	6.33	(A) 9.49†	39.90	1.27	(A) 4.43†	6.38
		GA	6.33	(G) 90.51		6.33	(G) 95.57	
		GG	87.34			92.41		
ABCB1	rs2235013	AA	17.72	(A) 48.10†	4.71	31.65	(A) 51.90	3.56
		GA	60.76	(G) 51.90		40.51	(G) 48.10†	
		GG	21.52			27.85		
ABCB1	rs2235033	CC	17.72	(C) 48.10†	4.71	32.91	(C) 52.53	4.54
		TC	60.76	(T) 51.90		39.24	(T) 47.47†	
		TT	21.52			27.85		
ABCB1	rs3213619	CC	0.00	(C) 1.27†	0.02	0.00	(C) 3.16†	0.11
		CT	2.53	(T) 98.73		6.33	(T) 96.84	
		TT	97.47			93.67		
ABCB1	rs9282564	AA	91.14	(A) 95.57	0.21	84.81	(A) 91.14	4.68
		AG	8.86	(G) 4.43†		12.66	(G) 8.86†	
		GG	0.00			2.53		
ABCC2	rs3740066	CC	34.18	(C) 58.86	0.04	37.97	(C) 62.66	0.30
		CT	49.37	(T) 41.14†		49.37	(T) 37.34†	
		TT	16.46			12.66		
ABCC2	rs2273697	AA	6.33	(A) 20.25†	1.90	2.53	(A) 19.62†	0.70
		GA	27.85	(G) 79.75		34.18	(G) 80.38	
		GG	65.82			63.29		
ABCC2	rs717620	CC	59.49	(C) 77.85	0.41	63.29	(C) 78.48	1.01
		TC	36.71	(T) 22.15†		30.38	(T) 21.52†	
		TT	3.80			6.33		
ABCG2	rs2231137	AA	0.00	(A) 6.33†	0.46	1.27	(A) 7.59†	0.96
		GA	12.66	(G) 93.67		12.66	(G) 92.41	
		GG	87.34			86.08		
ABCG2	rs2231142	AA	0.00	(A) 6.33†	0.46	1.27	(A) 5.06†	4.41
		CA	12.66	(C) 93.67		7.59	(C) 94.94	
		CC	87.34			91.14		
CYP3A4	rs2740574	AA	97.47	(A) 98.73	0.02	83.54	(A) 91.14	0.35
		GA	2.53	(G) 1.27†		15.19	(G) 8.86†	
		GG	0.00			1.27		
CYP3A5	rs10264272	AA	0.00	(A) 0.00	NA	0	(A) 0.63†	0.00

		AG	0.00	(G) 100		1.27	(G) 99.37	
		GG	100			98.73		
CYP3A5	rs776746	AA	1.27	(A) 8.23†	0.61	1.27	(A) 11.39†	0.00
		GA	13.92	(G) 91.77		20.25	(G) 88.61	
		GG	84.81			78.48		
CYP2B6	rs2279343	AA	45.57	(A) 67.72	0.02	53.16	(A) 72.78	0.01
		GA	44.30	(G) 32.28†		39.24	(G) 27.22†	
		GG	10.13			7.59		
CYP2B6	rs3745274	GG	0.00	(G) 43.67†	60.11	0.00	(G) 40.51†	46.36
		GT	87.34	(T) 56.33		81.01	(T) 59.49	
		TT	12.66			18.99		
CYP2C9	rs1799853	CC	79.75	(C) 89.87	1.27	77.22	(C) 88.61	1.65
		CT	20.25	(T) 10.13†		22.78	(T) 11.39†	
		TT	0.00			0.00		
CYP2C9	rs1057910	AA	92.41	(A) 96.20	0.16	87.34	(A) 91.77	17.08
		CA	7.59	(C) 3.80†		8.86	(C) 8.23†	
		CC	0.00			3.80		
CYP2C19	rs4244285	AA	2.53	(A) 16.46†	0.02	3.80	(A) 13.92†	2.40
		GA	27.85	(G) 83.54		20.25	(G) 86.08	
		GG	69.62			75.95		
MTHFR	rs1801131	AA	49.37	(A) 68.99	0.69	64.56	(A) 81.01	0.49
		CA	39.24	(C) 31.01†		32.91	(C) 18.99†	
		CC	11.39			2.53		
MTHFR	rs1801133	CC	44.30	(C) 65.82	0.19	21.52	(C) 53.80	8.92
		TC	43.04	(T) 34.18†		64.56	(T) 46.20†	
		TT	12.66			13.92		
NOD2	rs2066844	CC	88.61	(C) 94.30	0.36	91.14	(C) 95.57	0.21
		CT	11.39	(T) 5.70†		8.86	(T) 4.43†	
		TT	0.00			0.00		
NOD2	rs2066845	CC	0.00	(C) 1.90†	0.04	0.00	(C) 1.90†	0.04
		CG	3.80	(G) 98.10		3.80	(G) 98.10	
		GG	96.20			96.20		
TPMT	rs1142345	AA	88.61	(A) 94.30	0.36	93.67	(A) 96.84	0.11
		GA	11.39	(G) 5.70†		6.33	(G) 3.16†	
		GG	0.00			0.00		
TPMT	rs1800460	AA	0.00	(A) 5.06†	0.28	0.00	(A) 2.53†	0.07
		GA	10.13	(G) 94.94		5.06	(G) 97.47	
		GG	89.87			94.94		
TPMT	rs1800462	CC	0.00	(C) 1.90†	0.04	0.00	(C) 1.90†	0.04
		GC	3.80	(G) 98.10		3.80	(G) 98.10	
		GG	96.20			96.20		
SLCO1A2	rs11568563	AA	81.01	(A) 90.51	1.10	83.54	(A) 91.77	0.80
		CA	18.99	(C) 9.49†		16.46	(C) 8.23†	
		CC	0.00			0.00		
SLCO1B1	rs2306283	AA	54.43	(A) 70.89	4.11	40.51	(A) 63.92	0.02
		AG	32.91	(G) 29.11†		46.84	(G) 36.08†	
		GG	12.66			12.66		
SLCO1B1	rs4149056	CC	1.27	(C) 13.92†	0.32	3.80	(C) 15.19†	1.34
		TC	25.32	(T) 86.08		22.78	(T) 84.81	
		TT	73.42			73.42		
UGT1A9	rs6714486	AA	0.00	(A) 5.70†	0.36	0.00	(A) 4.43†	0.21
		TA	11.39	(T) 94.30		8.86	(T) 95.57	
		TT	88.61			91.14		
UGT1A9	rs7251330	CC	0.00	(C) 3.80†	0.16	0.00	(C) 4.43†	0.21
		TC	7.59	(T) 96.20		8.86	(T) 95.57	
		TT	92.41			91.14		

†MAF (minor allele frequency); χ^2: chi-square test result (Hardy Weinberg equilibrium while $\chi^2 < 5.99$).
